# Prevalence of depressive symptoms among intensive care unit nurses: a systematic review and meta-analysis of observational studies

**DOI:** 10.3389/fpubh.2026.1956872

**Published:** 2026-10-30

**Authors:** Cheng-Xue Huang, Quan-Xiu Tang, Yan Lu, Yue Ruan, Xia Li

**Affiliations:** 1 Department of Critical Care Medicine, West China Hospital, Sichuan University, Chengdu, Sichuan, China; 2 China/West China School of Nursing, Sichuan University, Chengdu, Sichuan, China

**Keywords:** depressive symptoms, intensive care unit, meta-analysis, nurses, prevalence

## Abstract

**Background:**

Despite the extensive body of literature on the prevalence of depressive symptoms among intensive care unit (ICU) nurses, the reported rates have exhibited marked inconsistency across studies. This meta-analysis aimed to determine the pooled prevalence of depressive symptoms and to explore potential sources of between-study variation in ICU nurses.

**Methods:**

A systematic search of PubMed, Web of Science, Scopus, Embase, Cochrane Library, CINAHL, and PsycINFO was conducted from database inception to September 30, 2026. The pooled prevalence of depressive symptoms was calculated using a random-effects model, with heterogeneity evaluated using the I^2^ statistic. Subgroup analyses and meta-regression were employed to identify the potential sources of heterogeneity.

**Results:**

A total of 38 studies comprising 22,474 ICU nurses representing 21 countries were included. The pooled prevalence of depressive symptoms in ICU nurses was 38.6% (95% CI: 31.2–46.3%, 95% PI: 2.8–84.6%, *I*^2^ = 98.9%, τ^2^ = 0.23). In exploratory univariate and multivariable meta-regression analyses, economic status and data collection period were associated with the prevalence of depressive symptoms among ICU nurses (all *p* < 0.05).

**Conclusion:**

Although the depressive symptoms among ICU nurses are common, the substantial heterogeneity and the wide 95% PI indicate that the true prevalence varies considerably across settings. This pooled estimate should therefore not be interpreted as a stable global prevalence. Instead, the findings highlight the marked variability in the prevalence of depressive symptoms across populations, economic contexts, and data collection periods. These findings highlight the need for increased attention to ICU nurses’ mental health and for context-sensitive organizational support to promote long-term psychological well-being in this high-risk population.

## 1 Introduction

The occupational mental health of intensive care unit (ICU) nurses has emerged as a growing global healthcare priority (1). In contrast to general nursing units, the ICU presents a uniquely challenging work environment. ICU nurses are tasked with managing critically ill patients and are routinely exposed to a range of occupational stressors, such as high patient acuity, recurrent encounters with mortality, complex ethical dilemmas surrounding end-of-life care, and heightened vulnerability to workplace violence (2, 3). The accumulation of occupational stressors exerts a compounding effect on ICU nurses, heightening their susceptibility to adverse psychological outcomes, notably depressive symptoms, anxiety, and sleep impairment (4–6). Depressive symptoms are clinically characterized by enduring depressed mood, anhedonia, and diminished interest, frequently accompanied by fatigue, sleep and appetite dysregulation, attentional deficits, low self-appraisal, and, in severe instances, suicidal ideation (7). Previous literature has demonstrated that depressive symptoms impair ICU nurses’ quality of life and professional performance and are also associated with increased medical errors, compromised patient safety, and heightened turnover intention, ultimately undermining the quality of critical care services (8–10). Given the above considerations, conducting a comprehensive assessment of the prevalence of depressive symptoms in ICU nurses and identifying its moderating factors is essential for formulating evidence-based organizational policies aimed at promoting occupational mental health.

In recent years, a large number of observational studies have investigated the prevalence of depressive symptoms among ICU nurses using various self-report assessment instruments (10–12). However, the reported estimates have varied considerably across studies, ranging from 6.1 to 77.9% (10–12). These discrepancies may be attributable to multiple factors, including economic status, cultural background, sample size, timing of data collection, age distribution, and work experience. This substantial heterogeneity prevents both an accurate estimate of depressive symptoms burden in ICU nurses and the generalizability of results to other healthcare settings. To date, although a previous meta-analysis has synthesized the available evidence (13), it did not include studies conducted after the outbreak of the COVID-19 pandemic, a period that may have profoundly influenced the mental health of ICU nurses. Moreover, that study did not perform sufficient subgroup analyses or meta-regression to systematically explore the sources of between-study heterogeneity. Therefore, an updated and more comprehensive synthesis is warranted to provide a robust prevalence estimate and to identify key moderators of depressive symptoms in this population. To address these gaps, we conducted a meta-analysis of observational studies to determine the pooled prevalence of depressive symptoms and identify its potential moderating factors in ICU nurses.

## 2 Methods

This meta-analysis of observational studies was performed in accordance with the Preferred Reporting Items for Systematic Reviews and Meta-Analyses (PRISMA) guidelines (14), and its protocol was prospectively registered with PROSPERO (CRD420261435951).

### 2.1 Search strategy

We systematically searched PubMed, Web of Science, Scopus, Embase, the Cochrane Library, CINAHL, and PsycINFO from their respective dates of inception to September 30, 2026, limiting our search to English-language articles. The search strategy (Supplementary Table S1) was constructed using Boolean operators to combine controlled vocabulary (MeSH) and free-text terms, such as nurses, nursing staff, intensive care units, depressive symptoms, and depression. The reference lists of all included studies were also manually examined to uncover additional studies that might meet the eligibility criteria.

### 2.2 Eligibility criteria

Eligibility criteria were defined as follows: (1) participants were nurses working in intensive care units, including adult, pediatric, and neonatal ICUs; (2) the study design was cross-sectional or longitudinal; (3) depressive symptoms were assessed using a validated instrument; (4) the prevalence of depressive symptoms was either reported directly or could be derived from the available data. Publications were excluded if they were conference abstracts, protocols, case reports, reviews, duplicates, or articles not published in English. To identify potentially overlapping samples, we compared publications by author, setting, recruitment period, and sample characteristics. When overlap was suspected, we retained only the report with the largest and most complete sample, or the earliest publication if sample sizes were equal.

### 2.3 Study selection and data extraction

Data were selected and extracted independently by two reviewers, with disagreements adjudicated through discussion with a third reviewer. The following details were collected from each study: first author, publication year, country, study design, sample size, mean age, proportion of married participants, proportion of female participants, assessment tool, and depressive symptoms prevalence.

### 2.4 Risk of bias assessment

Risk of bias was evaluated independently by two reviewers using the Joanna Briggs Institute (JBI) checklist for prevalence studies (15), which comprises nine assessment items. Each item received a score of “yes” (indicating low risk), “no” (high risk), or “unclear/not applicable.” On the basis of the percentage of “yes” responses, studies were classified into low (≥ 70%), moderate (50–69%), or high (≤ 49%) risk-of-bias categories (15). Any differences in scoring were settled via discussion with a third reviewer to reach agreement.

### 2.5 Statistical analysis

Statistical analyses were conducted with Stata 19.0. The Freeman-Tukey double-arcsine transformation was applied to the prevalence data before the main meta-analysis in order to stabilize the variance of proportions, particularly given the presence of studies with very small or very large prevalence estimates and marked differences in sample size across studies. Pooled estimates were then back-transformed to the original proportion scale using the inverse Freeman-Tukey transformation, so that the results could be interpreted as prevalence estimates. A random-effects model was chosen *a priori* because clinical and methodological heterogeneity across studies was expected. We evaluated between-study heterogeneity using the I^2^ statistic and tau-squared (τ^2^), with τ^2^ estimated using the restricted maximum likelihood (REML) method, and adopted a threshold of *I*^2^ ≥ 50% to denote substantial heterogeneity. The 95% prediction interval (PI) was also derived to forecast the expected range of true prevalence in future settings. Subgroup analyses were carried out according to economic status, data collection period, and assessment instrument to identify possible sources of heterogeneity. For these analyses, countries were classified as developed or developing according to the United Nations M49 (2022) classification of developed and developing regions (16). Data collection period was classified as “during the COVID-19 outbreak” if it occurred between 11 March 2020 (when WHO declared COVID-19 a pandemic) and 5 May 2023 (when WHO determined that COVID-19 no longer constituted a Public Health Emergency of International Concern), and as “outside the COVID-19 outbreak” if it occurred outside this period. For studies whose data collection spanned both periods, the classification was based on the period during which the majority of data were collected. For studies that did not report the data-collection period, the subgroup was determined based on the publication year. In addition, depression scales were classified as depression-specific if they were designed primarily to measure depressive symptoms, and as non-depression-specific if they measured general psychological distress or multiple psychological dimensions rather than depression alone. Exploratory univariate meta-regression was additionally performed to evaluate the relationship between the pooled prevalence and covariates such as sample size, mean age, percentage of females, proportion of married individuals, and the aforementioned subgroup variables. An exploratory multivariable meta-regression based on complete cases was also conducted. Covariates were selected *a priori* based on clinical plausibility, and only variables with sufficient available data were included. Meta-regression coefficients were estimated on the Freeman-Tukey transformation scale and are reported as such. Publication bias was examined by visual inspection of funnel plots and quantitatively tested with Egger’s test. The trim-and-fill analysis was performed to identify and impute potentially missing studies and to evaluate the robustness of the pooled estimate. The stability of the results was further assessed using a leave-one-out sensitivity analysis. In addition, we performed sensitivity analyses to examine whether the pooled estimate was influenced by methodological features, including excluding studies with moderate risk of bias, excluding studies using non-depression-specific instruments, excluding studies with unclear thresholds, and excluding the single largest study. We also performed a sensitivity analysis using a logit-transformed random-effects model to assess whether the pooled prevalence estimate was robust to the choice of meta-analytic method. Statistical significance was set at two-sided *p* < 0.05.

## 3 Results

### 3.1 Literature selection and characteristics

The database search initially generated 2,660 citations. After eliminating duplicate entries and studies not relevant to the research question, 38 original articles remained for meta-analysis (Figure 1). These investigations, all of which utilized a cross-sectional design, involved a total of 22,474 ICU nurses representing 21 countries, most of which are classified as developing economies. The prevalence of depressive symptoms in ICU nurses varied between 6.1 and 77.9%. A full description of the included studies is available in Table 1.

**Figure 1:**
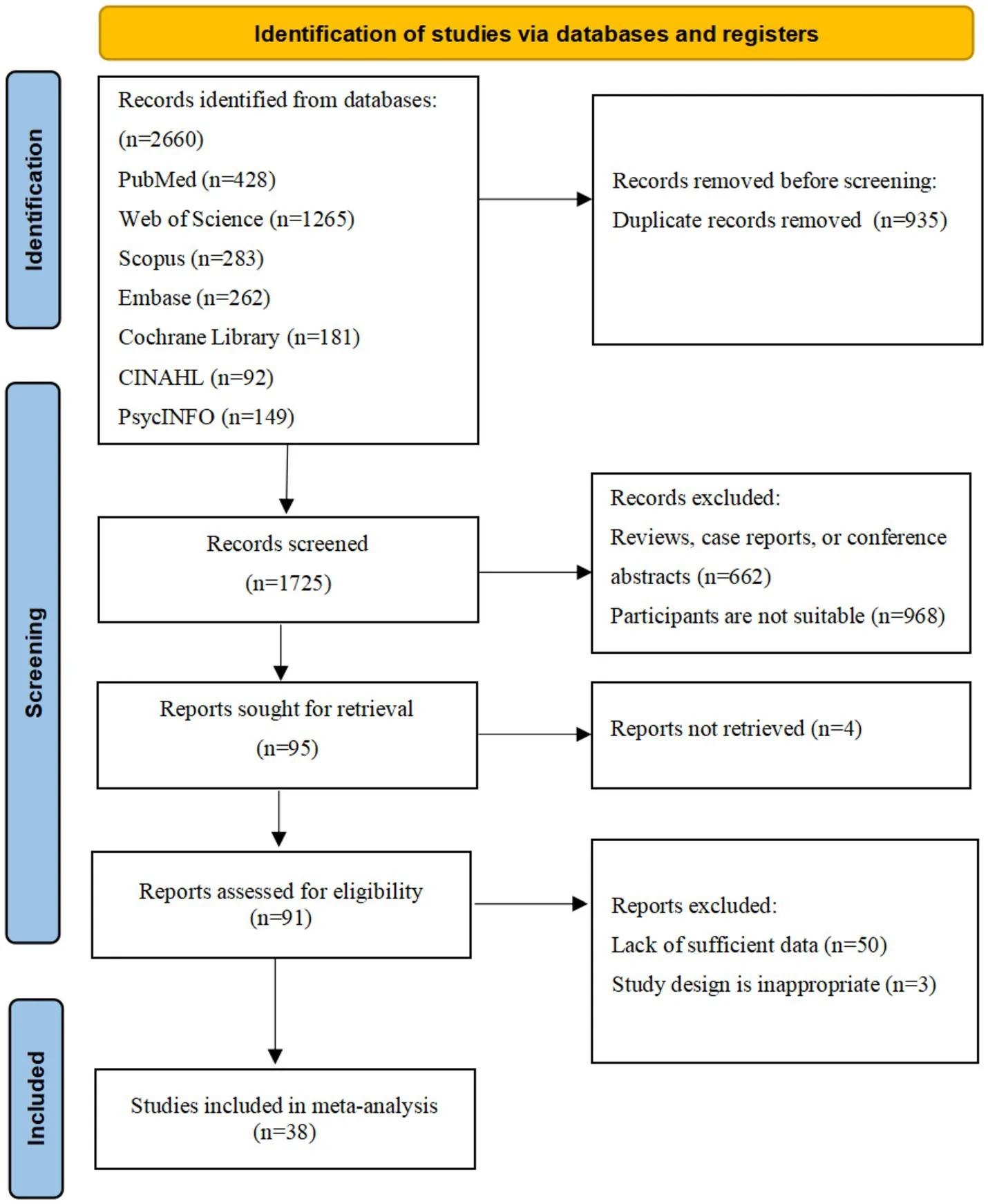
Flow diagram of study selection.

**Table 1:** Characteristics of included studies.

Study	Country	Data collection period	Economic status	Study design	Sample size	Married (%)	Mean age (years)	Female (%)	Assessment tool	Cut-off	Prevalence (%)
Alkubati et al. (10)	Saudi Arabia	April–June 2023	Developing country	Cross-sectional	262	56.1	NA	43.9	HADS	8	44.2
Anan et al. (27)	Jordan	June–August 2022	Developing country	Cross-sectional	260	53.5	30.8	55.8	DASS-42	10	61.9
Baraka et al. (28)	Egypt	During the COVID-19 outbreak	Developing country	Cross-sectional	200	67	NA	61.5	DASS-21	NA	52.5
Bizhanpour Sarabi et al. (11)	Iran	2024–2025	Developing country	Cross-sectional	389	50.6	37.6	60.4	BDI-II	14	77.9
Bjorvatn et al. (29)	Norway	October 2005–January 2006	Developed country	Cross-sectional	150	66.7	39.4	90.7	HADS	8	14.8
Bursch et al. (12)	USA	NA	Developed country	Cross-sectional	115	NA	NA	94.8	PHQ	NA	6.1
Cecere et al. (30)	Italy	February–May 2014	Developed country	Cross-sectional	140	57.1	41.8	46.4	DASS-21	5	10.7
Çelik et al. (20)	Turkey	December 2021–March 2022	Developing country	Cross-sectional	102	53.9	29.2	76.5	HADS	8	57.8
Crowe et al. (31)	Canada	May–June 2021	Developed country	Cross-sectional	425	NA	NA	92.5	DASS-21	10	69.6
de Vargas and Dias. (32)	Brazil	NA	Developing country	Cross-sectional	67	49	25	55	BDI	15	28.4
dos Santos Moura et al. (33)	Brazil	April–June 2016	Developing country	Cross-sectional	72	54.2	NA	52.8	BDI	NA	45.8
Guttormson et al. (25)	USA	October 2020–January 2021	Developed country	Cross-sectional	357	NA	NA	NA	PHQ	5	44.6
Heesakkers et al. (34)	Netherlands	September 2020	Developed country	Cross-sectional	726	NA	45	73.8	HADS	8	18.6
Hwang and Lee. (35)	Korea	During the COVID-19 outbreak	Developed country	Cross-sectional	131	15.3	28.7	90.1	CES-D	16	44.3
Ismail et al. (36)	Saudi Arabia	January–April 2023	Developing country	Cross-sectional	87	51.7	37	100	DASS-21	14	46.0
Janda et al. (37)	Czech Republic	NA	Developed country	Cross-sectional	151	36.4	31.2	91	HADS	NA	14.6
Jasper et al. (38)	Germany	NA	Developed country	Cross-sectional	89	NA	NA	NA	HADS	8	10.1
Kandemir et al. (39)	Turkey	July 2020	Developing country	Cross-sectional	194	37.6	30	71.1	DASS-21	NA	75.8
Karadağ and Çiçek. (40)	Turkey	December 2020–March 2021	Developing country	Cross-sectional	304	47	30.6	80.6	HADS	8	76.6
Kc et al. (19)	Nepal	During the COVID-19 outbreak	Developing country	Cross-sectional	113	31	NA	99.1	BDI	15	41.6
Kumar et al. (41)	India	December 2019	Developing country	Cross-sectional	125	33.6	NA	83.2	PHQ-2	NA	24.0
Li et al. (42)	China	During the COVID-19 outbreak	Developing country	Cross-sectional	78	64.1	NA	82.1	SDS	53	44.9
Li et al. (43)	China	December 2023–January 2024	Developing country	Cross-sectional	13,745	58.9	NA	84.2	PHQ-9	5	57.9
Lu et al. (44)	China	March 2016–April 2017	Developing country	Cross-sectional	500	51.2	27.7	91.8	CES-D	16	56.0
Mathew and Mathew. (45)	India	June 2022	Developing country	Cross-sectional	383	51.2	33.8	84	HADS	8	28.0
Mealer et al. (46)	USA	NA	Developed country	Cross-sectional	742	67.5	43.6	90.6	HADS	8	11.3
Petrișor et al. (47)	Romania	October 2020–February 2021	Developed country	Cross-sectional	79	65.8	37.1	89.9	PHQ-4	3	26.6
Rawas et al. (48)	Saudi Arabia	May–August 2021	Developing country	Cross-sectional	276	NA	NA	88.7	BDI-II	14	55.4
Sawalma et al. (49)	Palestine	March–May 2023	Developing country	Cross-sectional	273	53.8	29	35.5	DASS-21	NA	29.7
Swapnil et al. (50)	India	NA	Developing country	Cross-sectional	68	72.5	29	100.0	GHQ-28	NA	14.7
Tajvar et al. (51)	Iran	2013	Developing country	Cross-sectional	56	37.4	31.6	100	GHQ-28	NA	10.7
Tamrakar et al. (52)	Nepal	October–November 2020	Developing country	Cross-sectional	96	38.5	27.4	NA	HADS	8	39.6
Tatsuno et al. (53)	Japan	November–December 2020	Developed country	Cross-sectional	334	NA	37.4	64.4	HADS	8	56.2
Vasconcelos et al. (54)	Brazil	July 2014	Developing country	Cross-sectional	91	37.4	30.8	89	BDI	15	11.0
Wu et al. (55)	China	December 2022	Developing country	Cross-sectional	137	NA	34	92.7	HADS	8	75.9
Yıldız. (56)	Turkey	September 2019–February 2020	Developing country	Cross-sectional	164	53	29.7	68.3	BDI	13	44.5
Yousif and Al-Fayyadh. (57)	Iraq	January–March 2024	Developing country	Cross-sectional	377	52.5	29.1	57.8	DASS-21	NA	15.9
Zhang et al. (58)	China	December 2022–January 2023	Developing country	Cross-sectional	616	62.7	28	79.6	PHQ-9	5	64.1

BDI, Beck Depression Inventory; CES-D, Center for Epidemiologic Studies Depression Scale; DASS, Depression Anxiety Stress Scales; GHQ, General Health Questionnaire; HADS, Hospital Anxiety and Depression Scale; PHQ, Patient Health Questionnaire; SDS, Self-Rating Depression Scale; NA, Not Available.

### 3.2 The results of risk of bias assessment

According to the JBI critical appraisal tool, 30 (78.9%) studies were categorized as having low methodological quality concerns, while the remaining 8 (21.1%) were deemed to carry moderate risk. Across the individual quality domains, the sample frame was judged appropriate in the majority of studies (*n* = 20, 52.6%). However, all investigations employed convenience sampling strategies. Regarding sample size, 27 (71.1%) studies provided clear justification and adequate statistical power. Moreover, all studies demonstrated satisfactory performance across four critical domains: comprehensive description of participants and clinical settings, adequate data coverage in analyses, adoption of validated measurement instruments, and appropriate statistical procedures. As for response rates, 21 (55.3%) studies were deemed adequate.

### 3.3 Pooled prevalence of depressive symptoms in ICU nurses

Given the substantial heterogeneity (*I*^2^ = 98.9%, τ^2^ = 0.23), a random-effects model was used, estimating the pooled prevalence of depressive symptoms in ICU nurses at 38.6% (95% CI: 31.2–46.3%, 95% PI: 2.8–84.6%) (Figure 2).

**Figure 2:**
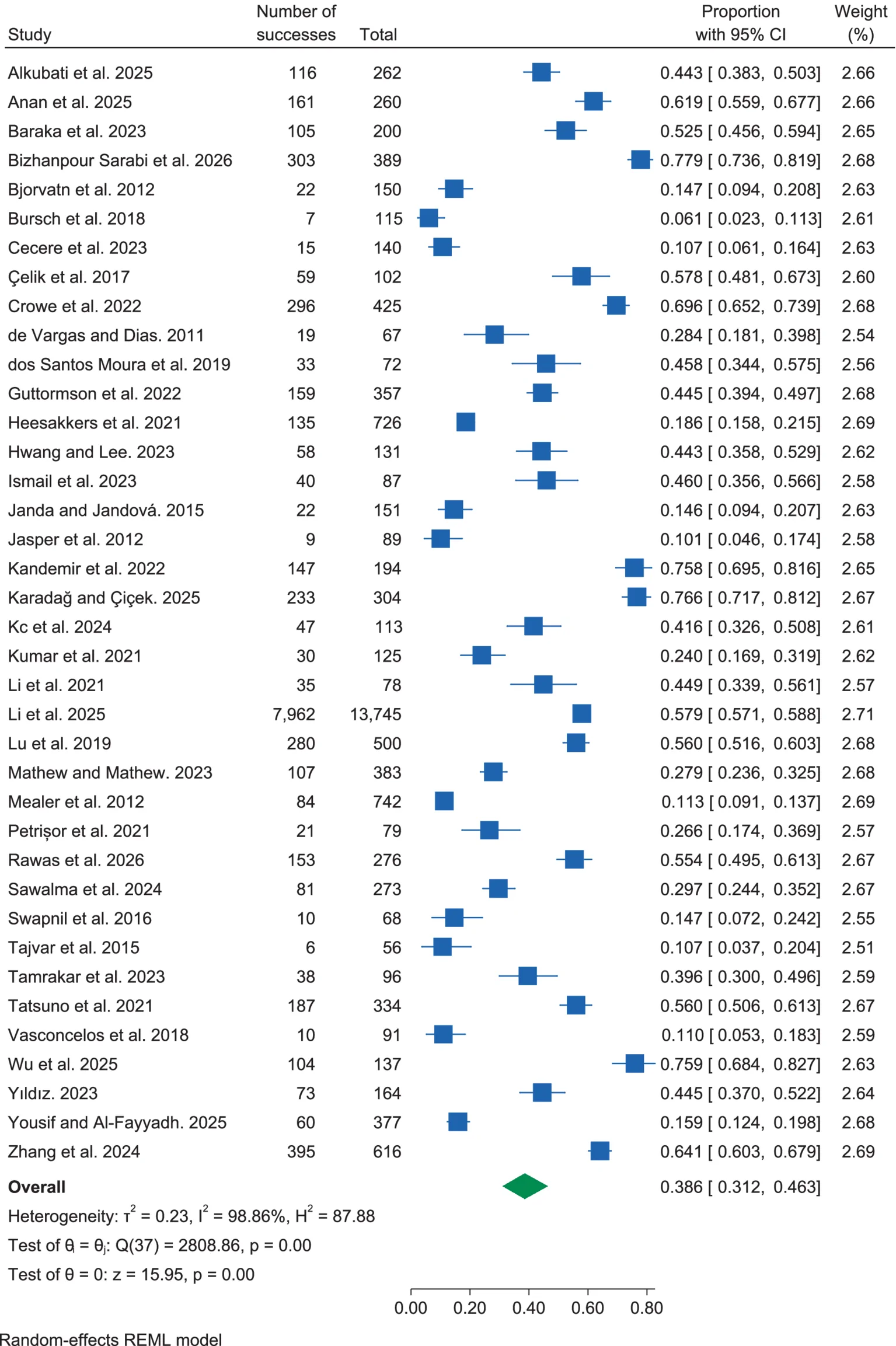
Forest plot of pooled prevalence of depressive symptoms in ICU nurses.

### 3.4 Subgroup analysis and meta-regression analysis

Subgroup analysis results are presented in Table 2. The prevalence of depressive symptoms among ICU nurses differed significantly based on the economic status of the countries where studies were conducted (*p* = 0.01) (Supplementary Figure S1). Specifically, a higher pooled estimate was observed in developing nations (45.1%, 95% CI: 36.8–53.7%) compared to their developed counterparts (25.4%, 95% CI: 14.6–38.0%). When stratified by data collection period (*p* = 0.01) (Supplementary Figure S2), studies carried out during the COVID-19 pandemic yielded a significantly higher prevalence (47.2%, 95% CI: 38.7–55.8%) relative to those performed outside this period (28.3%, 95% CI: 18.0–40.0%). By contrast, no statistically significant difference emerged when analyses were grouped according to the assessment instrument employed (*p* = 0.52) (Supplementary Figure S3): studies utilizing depressive symptoms-specific scales yielded a pooled prevalence of 41.5% (95% CI: 31.4–51.9%), whereas those adopting non-specific scales reported 36.6% (95% CI: 26.2–47.6%).

**Table 2:** Subgroup analyses of the pooled prevalence of depressive symptoms in ICU nurses.

Subgroups	Number of studies	Prevalence (95% CI)	Heterogeneity		*p*-values across subgroups
			I^2^	*P-*values	
Economic status					0.01
Developed countries	12	25.4% (14.6–38.0%)	98.4%	*p* < 0.001	
Developing countries	26	45.1% (36.8–53.7%)	98.5%	*P* < 0.001	
Data collection period					0.01
During the COVID‑19 outbreak	21	47.2% (38.7–55.8%)	97.5%	*P* < 0.001	
Outside the COVID‑19 outbreak	17	28.3% (18.0–40.0%)	99.0%	*P* < 0.001	
Assessment tool					0.52
Specific depressive symptoms scale	16	41.5% (31.4–51.9%)	98.5%	*P* < 0.001	
Non-specific depressive symptoms scale	22	36.6% (26.2–47.6%)	98.6%	*P* < 0.001	

Exploratory univariate meta-regression analysis (Table 3) demonstrated that economic status and data collection period were significantly associated with the pooled prevalence of depressive symptoms in ICU nurses (*p* < 0.05). In contrast, none of the other moderators examined, including sample size, mean age, proportion of married participants, proportion of female participants, and assessment tool, were significantly associated (all *p* > 0.05). In the exploratory multivariable meta-regression, both economic status and data collection period were independently associated with the pooled prevalence (all *p* < 0.05) (Table 4).

**Table 3:** Univariate meta-regression results of the pooled prevalence of depressive symptoms in ICU nurses.

Variables	Number of studies	Coefficient	Standard error	95%CI	*Z*-values	*P*-values	*R* ^2^
Sample size	38	3.20 × 10^−5^	3.60 × 10^−5^	(−0.00003, 0.0001)	0.89	0.375	0
Mean age	26	−0.0262	0.0189	(−0.0632, 0.0108)	−1.39	0.165	0
Proportion of married participants	30	−0.0020	00068	(−0.0152, 0.0112)	−0.30	0.766	0
Proportion of female participants	35	−0.0033	0.0048	(−0.0126, 0.0060)	−0.69	0.489	0
Economic status (reference: developed countries)							
Developing countries	26	0.4136	0.1576	(0.1047, 0.7246)	2.62	0.009	14.2
Data collection period (reference: during the COVID‑19 outbreak)							
Outside the COVID‑19 outbreak	17	−0.3879	0.1480	(−0.6779, −0.0979)	−2.62	0.009	13.8
Assessment tool (reference: specific depressive symptoms scale)							
Non-specific depressive symptoms scale	22	−0.0983	0.1614	(−0.4146, 0.2180)	−0.61	0.542	0

**Table 4:** Multivariable meta-regression results of the pooled prevalence of depressive symptoms in ICU nurses.

Variables	Number of studies	Coefficient	Standard error	95%CI	*Z*-values	*P*-values	*R* ^2^
Sample size	38	3.69 × 10^−5^	3.08 × 10^−5^	(−0.00002, 0.0001)	1.20	0.231	31.1
Economic status (reference: developed countries)							
Developing countries	26	0.4017	0.1433	(0.1209, 0.6826)	2.80	0.005	
Data collection period (reference: during the COVID‑19 outbreak)							
Outside the COVID‑19 outbreak	17	−0.4548	0.1371	(−0.7234, −0.1861)	−3.32	0.001	
Assessment tool (reference: specific depressive symptoms scale)							
Non-specific depressive symptoms scale	22	−0.1141	0.1391	(−0.3866, 0.1584)	−0.82	0.412	

### 3.5 Publication bias and sensitivity analysis

Visual inspection of the funnel plot (Figure 3) revealed asymmetry, and Egger’s regression test was statistically significant (Supplementary Figure S4) (*p* < 0.05). However, given the considerable between-study heterogeneity, this asymmetry may reflect small-study effects or other sources of variation and should not be interpreted as definitive evidence of publication bias. Consistent with this, the trim-and-fill analysis did not identify any missing studies (Supplementary Figure S5), and the pooled estimate was unchanged. The leave-one-out sensitivity analysis showed that no single study disproportionately influenced the pooled estimate (Supplementary Figure S6). In addition, sensitivity analyses excluding studies with moderate risk of bias (41.8%, 95%CI: 33.8–50.0%), studies using non-depression-specific instruments (41.5%, 95%CI: 31.4–51.9%), studies with unclear thresholds (42.1%, 95%CI: 33.9–50.5%), and the single largest study (38.1%, 95%CI: 30.5–45.9%), all yielded consistent results, with no substantial change in the pooled estimate (Supplementary Figures S7–S10). A sensitivity analysis using a logit-transformed random-effects model also produced a comparable pooled estimate (37.2%, 95%CI: 29.5–45.6%) (Supplementary Figure S11).

**Figure 3:**
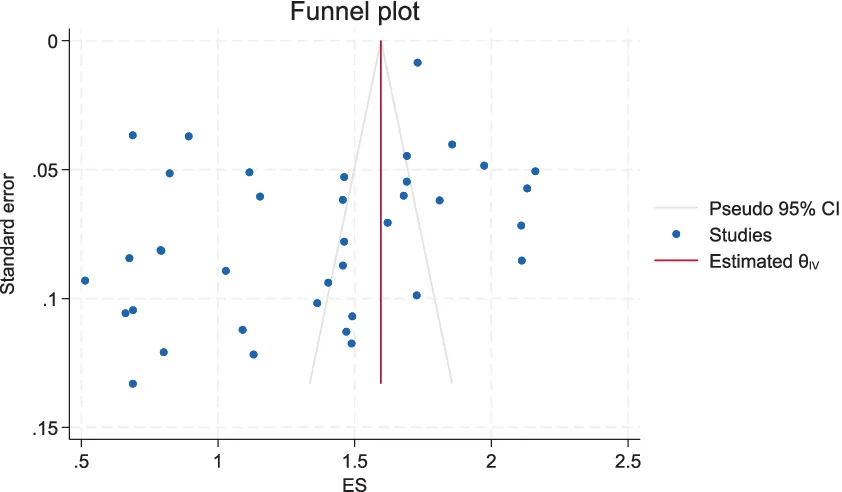
Funnel plot.

## 4 Discussion

This meta-analysis of observational studies was conducted to estimate the pooled prevalence of depressive symptoms among ICU nurses and to explore potential moderators that may account for between-study heterogeneity. Across 38 eligible studies, the pooled prevalence of depressive symptoms among ICU nurses was 38.6%, exceeding the figure reported in an earlier meta-analysis (13). This updated review extends the evidence period to 2026 and adds several analytical improvements, including subgroup analyses by economic status and data-collection period, univariate meta-regression, a leave-one-out sensitivity analysis, a sensitivity analysis excluding a highly influential study, and explicit reporting of the prediction interval and tau-squared. These additions provide a more comprehensive and transparent synthesis of the evidence and allow a more cautious and context-sensitive interpretation of the pooled prevalence than was possible in the previous meta-analysis. However, caution is advised when applying these findings to clinical nursing management, given the considerable heterogeneity (high I^2^ statistic and wide prediction interval), which indicates that the true prevalence may vary substantially across settings and that the pooled estimate should not be considered a stable global prevalence. This heterogeneity is likely to be partly attributable to the wide variation in the instruments and cut-offs used to define depressive symptoms across the included studies. In addition, 8 of the 38 included studies were categorized as having moderate methodological quality concerns, and all included studies used convenience sampling. These methodological limitations further reinforce that the pooled estimate should be interpreted as an approximate indication of the burden of depressive symptoms among ICU nurses rather than as a precise or internationally representative prevalence figure. The substantial burden of depressive symptoms observed among ICU nurses may be attributable to the multifaceted interactions between adverse workplace conditions, occupational stressors, and organizational policies. Nursing workforce shortages have consistently posed a formidable global public health concern (17). ICUs, in particular, tend to bear the brunt of understaffing, compelling ICU nurses to assume excessive workloads (18). Consequently, these nurses are subjected to prolonged physical and psychological stress, with inadequate opportunities for respite and recovery. Moreover, in contrast to general ward settings, ICUs are characterized by higher patient acuity, which inevitably exposes nurses to recurrent experiences of mortality and ethical challenges, including end-of-life care decisions and the administration of futile interventions (3). The compounding effects of repeated grief exposure and moral distress have been robustly identified as significant risk factors for depressive symptoms among ICU nursing staff (4). Finally, ICUs are regarded as having one of the highest prevalence rates of workplace violence across all healthcare departments. In the absence of robust organizational support mechanisms, such exposure compromises nurses’ psychological safety, engenders negative occupational appraisals, and potentiates depressive symptoms (19). These findings highlight the critical need for multi-level interventions addressing workplace stressors, organizational support, and mental health resources to mitigate depressive symptoms among ICU nurses and safeguard the sustainability of the critical care workforce.

Subgroup analyses and meta-regression revealed that economic status was significantly associated with the pooled prevalence of depressive symptoms among ICU nurses, with a substantially higher estimate observed in developing countries than in their developed counterparts. Several factors may plausibly contribute to the higher pooled prevalence of depressive symptoms observed among ICU nurses in developing countries, although these variables were not directly examined in the present meta-analysis and should therefore be regarded as possible explanations rather than established mechanisms. First, ICU nurse-to-patient ratios in these settings may often be suboptimal, potentially forcing nurses to shoulder excessive workloads with insufficient time for rest and recovery (20). Second, organizational support mechanisms, such as accessible psychological counseling and formal debriefing structures, may remain relatively underdeveloped (21, 22). Finally, socioeconomic factors, including lower remuneration, limited career advancement opportunities, and diminished professional recognition, may contribute to a pronounced effort-reward imbalance (11). If confirmed in future studies, these findings would highlight the need for targeted interventions to reduce depressive symptoms among ICU nurses in developing countries. Nursing managers and policymakers may consider workload reduction through improved staffing and shift scheduling, the establishment of accessible psychological support and formal debriefing services, and policy measures to enhance financial rewards, career progression, and professional recognition.

In this study, both subgroup analyses and exploratory meta-regression demonstrated that the data collection period was significantly associated with the pooled prevalence of depressive symptoms among ICU nurses. Specifically, studies that collected data during the COVID-19 outbreak reported a significantly higher prevalence than those conducted outside the outbreak period. This finding is consistent with previous studies reporting elevated psychological distress among healthcare workers during the COVID-19 pandemic (23). The COVID-19 pandemic represented a global public health crisis of unprecedented scale, placing extraordinary strain on healthcare systems internationally (19). As the outbreak escalated, many countries undertook rapid ICU capacity expansion in an effort to optimize patient throughput. This expansion, however, resulted in severely compromised nurse-to-patient ratios, substantially escalating the workload of frontline nurses and serving as a precursor to emotional disturbances (24). Moreover, during the initial stages of the pandemic, characterized by both high viral virulence and marked deficiencies in personal protective equipment, ICU nurses attending to COVID-19 patients faced heightened occupational exposure risks (25). The sustained perception of personal vulnerability, compounded by the escalating demands of critical care delivery, generated a persistent state of psychological distress, thereby increasing the likelihood of adverse emotional outcomes among this workforce (26).

## 5 Limitations

This study has several limitations. First, the restriction to English-language publications and the exclusion of grey literature may have introduced selection and publication bias, potentially affecting the pooled estimates. Second, the majority of the included studies were conducted in Asia, which may limit the generalizability of our findings to other geographic regions characterized by different cultural contexts, healthcare systems, and occupational environments. Third, the definition of depression was not sufficiently standardized across the included studies. A wide range of instruments was used, including the HADS, DASS, BDI, CES-D, PHQ, GHQ, and SDS, and even within the same instrument, versions, cut-off scores, and severity thresholds differed substantially, with many studies not reporting these details at all. This measurement heterogeneity may have influenced the pooled prevalence estimates. Fourth, in common with other meta-analyses of prevalence, substantial heterogeneity existed across studies, and the factors examined, such as economic status and data collection period, explained only a small proportion of this heterogeneity.

Finally, many demographic characteristics were inconsistently reported or missing across studies, which prevented further exploration of potential moderators. Therefore, future research should adopt standardized data reporting practices to facilitate data synthesis and meta-analytic integration.

## 6 Conclusion

Our findings indicate that depressive symptoms are common among ICU nurses. However, the extremely high heterogeneity and the wide 95% PI indicate that the actual prevalence varies substantially across settings. The pooled estimate should therefore not be interpreted as a stable global prevalence; rather, the prediction interval and the variability between populations, economic contexts, and data collection periods should be central to the interpretation of these findings. Therefore, hospital administrators should develop comprehensive organizational support strategies to alleviate psychological distress and promote occupational mental health.

## Data Availability

The original contributions presented in the study are included in the article/Supplementary material, further inquiries can be directed to the corresponding author.
