# Supplementary material for: Prevalence of depressive symptoms among intensive care unit nurses: a systematic review and meta-analysis of observational studies
Source: Front Public Health. 2026 Oct 30;14:1956872. doi: 10.3389/fpubh.2026.1956872 (PMC13759818; doi:10.3389/fpubh.2026.1956872)
Supplement: Supplementary file 1 [file Supplementary_file_1.docx]

**Supplemental Materials**

Table S1. The search strategies.

| Databases | Step | Search Strategies |
| --- | --- | --- |
| PubMed | #1 | "nurses"[Mesh Terms] |
|  | #2 | "nursing staff"[Mesh Terms] |
|  | #3 | "nurses"[Title/Abstract] OR "nursing staff"[Title/Abstract] OR "registered nurses"[Title/Abstract] OR "clinical nurses"[Title/Abstract] |
|  | #4 | "intensive care units"[MeSH Terms] |
|  | #5 | "critical care"[MeSH Terms] |
|  | #6 | "intensive care units"[Title/Abstract] OR "critical care"[Title/Abstract] OR "ICU"[Title/Abstract] |
|  | #7 | "depression"[Mesh Terms] |
|  | #8 | "depression"[Title/Abstract] OR "depressive symptoms"[Title/Abstract] OR "depressive disorder"[Title/Abstract] OR "mood disorder"[Title/Abstract] |
|  | #9 | #1 OR #2 OR #3 |
|  | #10 | #4 OR #5 OR #6 |
|  | #11 | #7 OR #8 |
|  | #12 | #9 AND #10 AND #11 |
| Web of Science | #1 | TS=(nurses) OR TS=(nursing staff) OR TS=(registered nurses) OR TS=(clinical nurses) |
|  | #2 | TS=(intensive care units) OR TS=(critical care) OR TS=(ICU) |
|  | #3 | TS=(depression) OR TS=(depressive symptoms) OR TS=(depressive disorder) OR TS=(mood disorder) |
|  | #4 | #1 AND #2 AND #3 |
| Scopus | #1 | TITLE-ABS-KEY("[nurses](https://www.ncbi.nlm.nih.gov/mesh/68009369)" OR "nursing staff" OR "registered nurses" OR "clinical nurses") |
|  | #2 | TITLE-ABS-KEY("intensive care units" OR "critical care" OR "ICU") |
|  | #3 | TITLE-ABS-KEY("depression" OR "depressive symptoms" OR "depressive disorder" OR "mood disorder") |
|  | #4 | #1 AND #2 AND #3 |
| Embase | **#1** | '[nurses](https://www.ncbi.nlm.nih.gov/mesh/68009369)'/exp |
|  | **#2** | 'nursing staff'/exp |
|  | **#3** | 'nurses':ti,ab,kw OR 'nursing staff':ti,ab,kw OR 'registered nurses':ti,ab,kw OR 'clinical nurses':ti,ab,kw |
|  | **#4** | 'intensive care units'/exp |
|  | #5 | 'intensive care units':ti,ab,kw OR 'critical care':ti,ab,kw OR 'ICU':ti,ab,kw |
|  | **#6** | 'depression'/exp |
|  | **#7** | **'depression':ti,ab,kw OR '**depressive symptoms**':ti,ab,kw OR '**depressive disorder**':ti,ab,kw OR '**mood disorder**':ti,ab,kw** |
|  | **#8** | #1 OR #2 OR #3 |
|  | **#9** | #4 OR #5 |
|  | **#10** | #6 OR #7 |
|  | **#11** | #8 AND #9 AND #10 |
| Cochrane Library | #1 | MeSH descriptor: [Nurses] explode all trees |
|  | #2 | **MeSH descriptor: [Nursing Staff] explode all trees** |
|  | #3 | (nurses):ti,ab,kw OR (nursing staff):ti,ab,kw OR (registered nurses):ti,ab,kw OR (clinical nurses):ti,ab,kw |
|  | #4 | MeSH descriptor: [Intensive Care Units] explode all trees |
|  | #5 | MeSH descriptor: [Critical Care] explode all trees |
|  | #6 | 'intensive care units':ti,ab,kw OR 'critical care':ti,ab,kw OR 'ICU':ti,ab,kw |
|  | #7 | MeSH descriptor: [depression] explode all trees |
|  | #8 | (depression):ti,ab,kw OR (depressive symptoms):ti,ab,kw OR (depressive disorder):ti,ab,kw OR (mood disorder):ti,ab,kw |
|  | #9 | #1 OR #2 OR #3 |
|  | #10 | #4 OR #5 OR #6 |
|  | #11 | #7 OR #8 |
|  | #12 | #9 AND #10 AND #11 |
| CINAHL | #1 | **MH Nurses OR MH Nursing Staff OR TI nurses OR AB nurses OR TI registered nurses OR AB registered nurses OR TI clinical nurses OR AB clinical nurses** |
|  | #2 | **MH Intensive Care Units OR MH Critical Care OR TI intensive care OR AB intensive care OR TI critical care OR AB critical care OR TI ICU OR AB ICU** |
|  | #3 | **MH Depression OR TI depression OR AB depression OR TI** depressive symptoms OR AB depressive symptoms OR **TI mood disorder OR AB mood disorder** |
|  | #4 | #1 AND #2 AND #3 |
| PsycINFO | #1 | **MH Nurses OR MH Nursing Staff OR TI nurses OR AB nurses OR TI registered nurses OR AB registered nurses OR TI clinical nurses OR AB clinical nurses** |
|  | #2 | **MH Intensive Care Units OR MH Critical Care OR TI intensive care OR AB intensive care OR TI critical care OR AB critical care OR TI ICU OR AB ICU** |
|  | #3 | **MH Depression OR TI depression OR AB depression OR TI** depressive symptoms OR AB depressive symptoms OR **TI mood disorder OR AB mood disorder** |
|  | #4 | #1 AND #2 AND #3 |

Table S2. Quality assessment results of included studies.

| Study | Q1 | Q2 | Q3 | Q4 | Q5 | Q6 | Q7 | Q8 | Q9 | Overall |
| --- | --- | --- | --- | --- | --- | --- | --- | --- | --- | --- |
| Alkubati et al. 2025 | Y | N | Y | Y | Y | Y | Y | Y | Y | L |
| Anan et al. 2025 | Y | N | Y | Y | Y | Y | Y | Y | Y | L |
| Baraka et al. 2023 | N | N | U | Y | Y | Y | Y | Y | N | M |
| Bizhanpour Sarabi et al. 2026 | Y | N | Y | Y | Y | Y | Y | Y | Y | L |
| Bjorvatn et al. 2012 | Y | N | Y | Y | Y | Y | Y | Y | N | L |
| Bursch et al. 2018 | N | N | U | Y | Y | Y | Y | Y | N | M |
| Cecere et al. 2023 | Y | N | Y | Y | Y | Y | Y | Y | N | L |
| Çelik et al. 2017 | Y | N | Y | Y | Y | Y | Y | Y | Y | L |
| Crowe et al. 2022 | N | N | Y | Y | Y | Y | Y | Y | U | M |
| de Vargas and Dias. 2011 | N | N | N | Y | Y | Y | Y | Y | N | M |
| dos Santos Moura et al. 2019 | N | N | N | Y | Y | Y | Y | Y | N | M |
| Guttormson et al. 2022 | N | N | Y | Y | Y | Y | Y | Y | U | L |
| Heesakkers et al. 2021 | N | N | Y | Y | Y | Y | Y | Y | Y | L |
| Hwang and Lee. 2023 | Y | N | Y | Y | Y | Y | Y | Y | Y | L |
| Ismail et al. 2023 | N | N | Y | Y | Y | Y | Y | Y | Y | L |
| Janda and Jandová. 2015 | Y | N | Y | Y | Y | Y | Y | Y | Y | L |
| Jasper et al. 2012 | N | N | U | Y | Y | Y | Y | Y | N | M |
| Kandemir et al. 2022 | Y | N | Y | Y | Y | Y | Y | Y | N | L |
| Karadağ and Çiçek. 2025 | N | N | Y | Y | Y | Y | Y | Y | U | L |
| Kc et al. 2024 | N | N | Y | Y | Y | Y | Y | Y | N | L |
| Kumar et al. 2021 | Y | N | Y | Y | Y | Y | Y | Y | Y | L |
| Li et al. 2021 | N | N | U | Y | Y | Y | Y | Y | Y | L |
| Li et al. 2025 | Y | N | Y | Y | Y | Y | Y | Y | Y | L |
| Lu et al. 2019 | Y | N | Y | Y | Y | Y | Y | Y | U | L |
| Mathew and Mathew. 2023 | Y | N | Y | Y | Y | Y | Y | Y | U | L |
| Mealer et al. 2012 | N | N | N | Y | Y | Y | Y | Y | N | M |
| Petrișor et al. 2021 | N | N | Y | Y | Y | Y | Y | Y | Y | L |
| Rawas et al. 2026 | Y | N | N | Y | Y | Y | Y | Y | Y | L |
| Sawalma et al. 2024 | Y | N | U | Y | Y | Y | Y | Y | Y | L |
| Swapnil et al. 2016 | N | N | U | Y | Y | Y | Y | Y | Y | L |
| Tajvar et al. 2015 | N | N | U | Y | Y | Y | Y | Y | U | M |
| Tamrakar et al. 2023 | N | N | U | Y | Y | Y | Y | Y | Y | L |
| Tatsuno et al. 2021 | Y | N | Y | Y | Y | Y | Y | Y | Y | L |
| Vasconcelos et al. 2018 | N | N | Y | Y | Y | Y | Y | Y | N | L |
| Wu et al. 2025 | Y | N | Y | Y | Y | Y | Y | Y | Y | L |
| Yıldız. 2023 | Y | N | Y | Y | Y | Y | Y | Y | Y | L |
| Yousif and Al-Fayyadh. 2025 | Y | N | Y | Y | Y | Y | Y | Y | Y | L |
| Zhang et al. 2024 | Y | N | Y | Y | Y | Y | Y | Y | Y | L |

Notes: Y: Yes, N: No, U: Unclear. L: Low risk, M: Moderate risk, H: High risk.

Q1: Was the sample frame appropriate to address the target population?

Q2: Were study participants sampled in an appropriate way?

Q3: Was the sample size adequate?

Q4: Were the study subjects and the setting described in detail?

Q5: Was the data analysis conducted with sufficient coverage of the identified sample?

Q6: Were valid methods used for the identification of the condition?

Q7: Was the condition measured in a standard, reliable way for all participants?

Q8: Was there appropriate statistical analysis?

Q9: Was the response rate adequate, and if not, was the low response rate managed appropriately?

Figure S1. The pooled prevalence of depressive symptoms in ICU nurses based on economic status.

Figure S2. The pooled prevalence of depressive symptoms in ICU nurses based on data collection period.

Figure S3. The pooled prevalence of depressive symptoms in ICU nurses based on assessment tool.

Figure S4. Egger's test results.

Figure S5. Trim-and-fill analysis

Figure S6. Leave-one-out sensitivity analysis.

Figure S7. Sensitivity analysis excluding studies with moderate risk of bias.

Figure S8. Sensitivity analysis excluding studies with the non-depression-specific instrument.

Figure S9. Sensitivity analysis excluding studies with unclear threshold.

Figure S10. Sensitivity analysis excluding the largest study.

Figure S11. Sensitivity analysis using a logit-transformed random-effects model.
